# Metabolomic analysis of plasma biomarkers in children with autism spectrum disorders

**DOI:** 10.1002/mco2.488

**Published:** 2024-02-28

**Authors:** Jun Liu, Yuhua Tan, Fan Zhang, Yan Wang, Shu Chen, Na Zhang, Wenjie Dai, Liqing Zhou, Ji‐Cheng Li

**Affiliations:** ^1^ Medical Research Center Yue Bei People's Hospital, Shantou University Medical College Shaoguan China; ^2^ Shaoguan Maternal and Child Health Hospital Shaoguan China; ^3^ Institute of Cell Biology Zhejiang University Hangzhou China; ^4^ Major Disease Biomarkers Research Laboratory School of Basic Medical Science, Henan University Kaifeng China

**Keywords:** autism spectrum disorder, biomarkers, machine learning, metabolomic, UPLC‐MS/MS

## Abstract

Autism spectrum disorder (ASD) presents a significant risk to human well‐being and has emerged as a worldwide public health concern. Twenty‐eight children with ASD and 33 healthy children (HC) were selected for the quantitative determination of their plasma metabolites using an ultraperformance liquid chromatography‐tandem mass spectrometry (UPLC‐MS/MS) platform. A total of 1997 metabolites were detected in the study cohort, from which 116 metabolites were found to be differentially expressed between the ASD and HC groups. Through analytical algorithms such as least absolute shrinkage selection operator (LASSO), support vector machine (SVM), and random forest (RF), three potential metabolic markers were identified as FAHFA (18:1(9Z)/9‐O‐18:0), DL‐2‐hydroxystearic acid, and 7(S),17(S)‐dihydroxy‐8(E),10(Z),13(Z),15(E),19(Z)‐docosapentaenoic acid. These metabolites demonstrated superior performance in distinguishing the ASD group from the HC group, as indicated by the area under curves (AUCs) of 0.935, 0.897, and 0.963 for the three candidate biomarkers, respectively. The samples were divided into training and validation sets according to 7:3. Diagnostic models were constructed using logistic regression (LR), SVM, and RF. The constructed three‐biomarker diagnostic model also exhibited strong discriminatory efficacy. These findings contribute to advancing our understanding of the underlying mechanisms involved in the occurrence of ASD and provide a valuable reference for clinical diagnosis.

## Graphical abstract

In this study, we collected a total of plasma from children with autism spectrum disorder (ASD) and normal healthy children (HC) after ethical committee approval and guardian consent, performed widely targeted metabolomics analysis, and identified a total of three potential metabolic biomarkers for distinguishing ASD from HC after bioinformatics analysis and multiple machine learning.

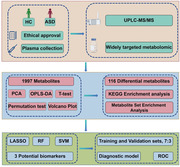

## 1 INTRODUCTION

Autism spectrum disorders (ASD) is a group of complex neurodevelopmental disorders characterized by social communication deficits, restrictive and repetitive behaviors or abnormal sensorimotor behaviors that have severe effects on individuals throughout their lives.^1^ Over the past 20 years, there has been an increasing trend in the prevalence of ASD globally, with the current prevalence reaching 0.62%–0.7%.^2^ In the United States, the prevalence of ASD surged from 5.8% to 25% from 2005 to 2016 and continues to rise.^3^ In China, the preliminary prevalence of ASD in the elementary school population is 7/1000, but the actual prevalence may be higher due to the lack of objective diagnostic criteria.^4^ Diagnosis currently relies heavily on subjective judgment and observation, leading to a lack of valid diagnostic markers.^5^ Early intervention has been shown to improve patients' symptoms and quality of life, highlighting the urgent need to develop rapid, efficient, and sensitive diagnostic biomarkers for ASD to identify and intervene early in children and improve their prognosis.

Recent studies have made progress in identifying potential peripheral blood biomarkers for ASD, including microRNA, LncRNA, and protein.^6^ For example, miR‐328‐3p and miR‐3135a are significantly downregulated in ASD children compared to controls, and serum levels of miR‐3135a and miR‐328‐3p can distinguish ASD children from healthy controls, indicating their potential as diagnostic biomarkers for ASD.^7^ Additionally, sAPP‐α levels were found to be significantly higher in children with ASD, and their expression levels in plasma correlated with disease severity, suggesting its potential as a biomarker to differentiate disease severity in children with ASD.^8^ Cortelazzo et al. employed proteomics to identify a set of 12 proteins associated with inflammation, which hold potential for diagnosing individuals with ASD.^9^ Shen et al. identified five proteins with significant potential for distinguishing between individuals with ASD and those in the control group.^10^ Furthermore, a multitude of studies have demonstrated the considerable translational potential of redox metabolism, cytokines, growth factors, neurotransmitters, and hormones in the diagnostic assessment of children with ASD.^11^ While these findings hold promise for the development of effective clinical diagnostic markers, further research is needed to fully understand the underlying mechanisms of ASD pathogenesis and identify more reliable diagnostic markers.

Metabolomics is an important component of systems biology, and with the advancement of metabolomics technologies, new technological platforms have emerged that are widely used to interpret the pathogenesis of various diseases and identify diagnostic markers.^12^, ^13^ In Alzheimer's disease, researchers have identified six differential metabolites and three major metabolic pathways through metabolomics.^14^ Similarly, in depression, metabolomic techniques have been used to identify differential metabolites and mechanisms of action for antidepressants.^15^ In the context of ASD, researchers have observed notable disparities in the abundance of metabolites present in the serum and urine of individuals with ASD compared to typically developing children.^16^, ^17^, ^18^ Using mass spectrometry techniques, Needham et al. successfully discerned that amino acids, lipids, and xenobiotic metabolites exhibit discriminative potential in distinguishing individuals with ASD from typically developing children.^19^ Tang et al. employed plasma proteomics and metabolomics techniques to ascertain that L‐glutamate and malate dehydrogenase possess discriminatory potential in distinguishing children with ASD from healthy children (HC), thereby suggesting their potential significance in the pathogenesis of ASD.^20^ However, the metabolic profile in children with ASD has yet to be fully explored, and whether metabolites can serve as diagnostic markers for ASD requires further investigation.

This study utilized an ultraperformance liquid chromatography‐tandem mass spectrometry (UPLC‐MS/MS) platform with widely targeted techniques to investigate metabolic disorders in children with ASD and identify potential molecular biomarkers of ASD in plasma via machine learning. By doing so, the study aimed to provide laboratory data that could enhance our understanding of the pathogenesis of ASD and contribute to the development of reliable diagnostic markers.

## 2 RESULTS

### 2.1 Clinical characteristics of the study population

In this study, a total of 61 individuals were included in the study population, with 28 children diagnosed with ASD and 33 healthy control children. Among the children with ASD, there were six females and 22 males. The distribution of ASD children according to birth order was as follows: 8 first borns, 14 second borns, 2 third borns, and 1 fourth born. The mean age of the children with ASD was 3.92 years. Of these children, 20 had a Childhood Autism Rating Scale score of mild, while eight had a score indicating moderate or severe autism. Importantly, there was no statistically significant difference in the composition of the study population between the healthy control and ASD groups (Table 1). This suggests that any differences observed between the metabolomic profiles of these two groups are likely associated with ASD status rather than other demographic factors such as age or sex.

**TABLE 1:** The study population's baseline clinical characteristics.

Characteristic	ASD	HC	*p*
Gender, *n* (%)	28	33	0.090 (chi‐square test)
Female	6 (9.8%)	15 (24.6%)	
Male	22 (36.1%)	18 (29.5%)	
Birth order, *n* (%)	25	29	0.699 (chi‐square test)
1	8 (14.8%)	13 (24.1%)	
2	14 (25.9%)	14 (25.9%)	
3	2 (3.7%)	2 (3.7%)	
4	1 (1.9%)	0 (0%)	
Age, median (IQR)	3.92 (2.79, 4.5)	3.42 (3.17, 5)	0.800 (Wilcoxon test)
CARS score, *n* (%)	28		
Mild (30–36)	20 (71.4%)	NA	
Moderate, severe (≥37)	8 (28.6%)	NA	

Abbreviations: ASD, autism spectrum disorder; CARS, Childhood Autism Rating Scale; HC, healthy control.

### 2.2 Metabolomic profiling and data quality assurance

**FIGURE 1:**
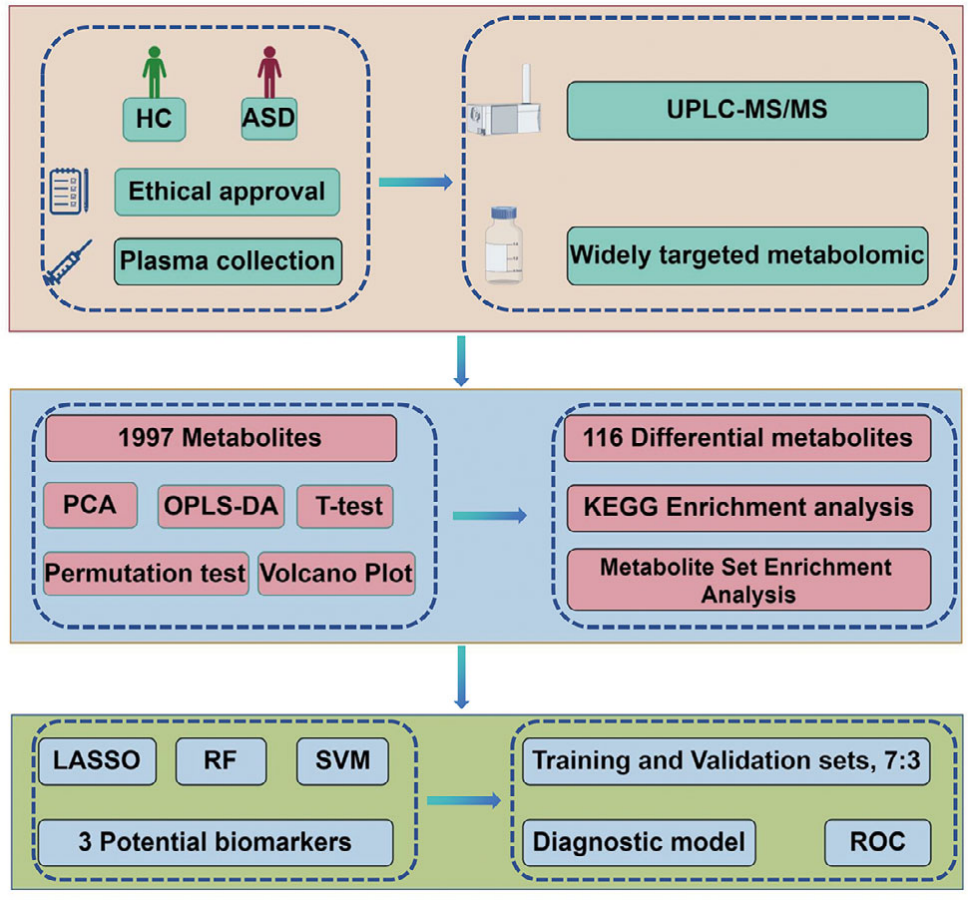
The flowchart graph of this study. ASD, autism spectrum disorder; HC, healthy control; KEGG, Kyoto Encyclopedia of Genes and Genomes; LASSO, least absolute shrinkage selection operator; OPLS‐DA, orthogonal partial least squares discriminant analysis; PCA, principal component analysis; RF, random forest; ROC, receiver operating characteristic; SVM, support vector machine; UPLC‐MS/MS, ultraperformance liquid chromatography‐tandem mass spectrometry.

**FIGURE 2:**
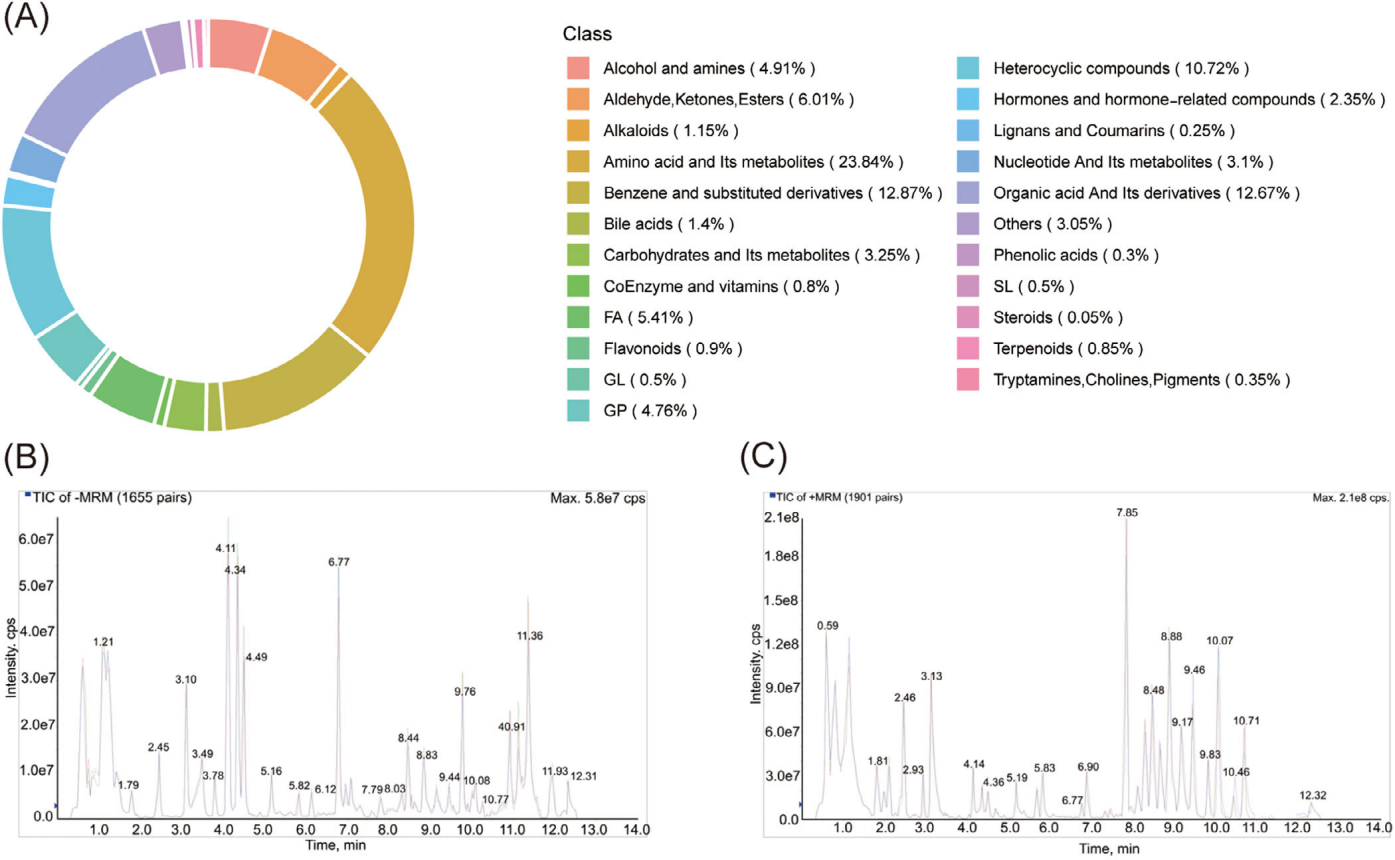
Composition of metabolites and total ionogram of quality control (QC) specimens. (A) Subclass composition of metabolites: This figure provides a comprehensive overview of the different subclasses of metabolites detected in our study. It includes detailed information on various metabolite classes, and each subclass is color‐coded for easy identification and understanding. (B) Total ion flow diagram of a QC specimen in positive ion mode: This diagram depicts the total ion flow observed in a QC specimen when analyzed in positive ion mode. It showcases the distribution and intensity of ions detected across the mass range. Peaks corresponding to different metabolites are labeled, allowing for the identification and tracking of specific compounds. (C) Total ion flow diagram of a QC specimen in negative ion mode: This diagram presents the total ion flow observed in a QC specimen when analyzed in negative ion mode. Similarly to Figure 1B, it illustrates the distribution and intensity of ions detected across the mass range. Peaks representing various metabolites are labeled for easier identification and comparison between positive and negative ion modes.

In order to investigate the metabolomic profiles of individuals diagnosed with ASD in contrast to a control group consisting of healthy individuals, the UPLC‐MS/MS platform was employed alongside comprehensive targeted metabolomic techniques for the purpose of metabolite identification and analysis. The study process was schematically presented in the flowchart (Figure 1). A total of 1997 metabolites were detected in each of the two groups. These metabolites were categorized into 23 subclasses, with amino acids and their metabolites, benzene and substituted derivatives, organic acids and their derivatives, and heterocyclic compounds accounting for 23.84%, 12.87%, 12.67%, and 10.72%, respectively (Figure 2A). To ensure the reliability and reproducibility of the data, quality control (QC) samples were employed. Total ion plots for each mixed QC sample were plotted and superimposed. The results showed a high degree of overlap among QC samples detected at different time points (Figure 2B,C). This indicates the high stability and reproducibility of the detection instrument used in this study. Based on the consistency observed in the QC samples, any differences observed between the metabolomic profiles of the ASD and healthy control groups are likely associated with biological differences rather than technical variability. This strengthens the confidence in the observed differences between the two groups. In summary, the metabolomics data obtained in this study demonstrate the robustness and accuracy of the analytical methods employed and provide valuable insights into the metabolic differences between ASD and healthy control individuals.

### 2.3 Differential metabolite analysis and identification

**FIGURE 3:**
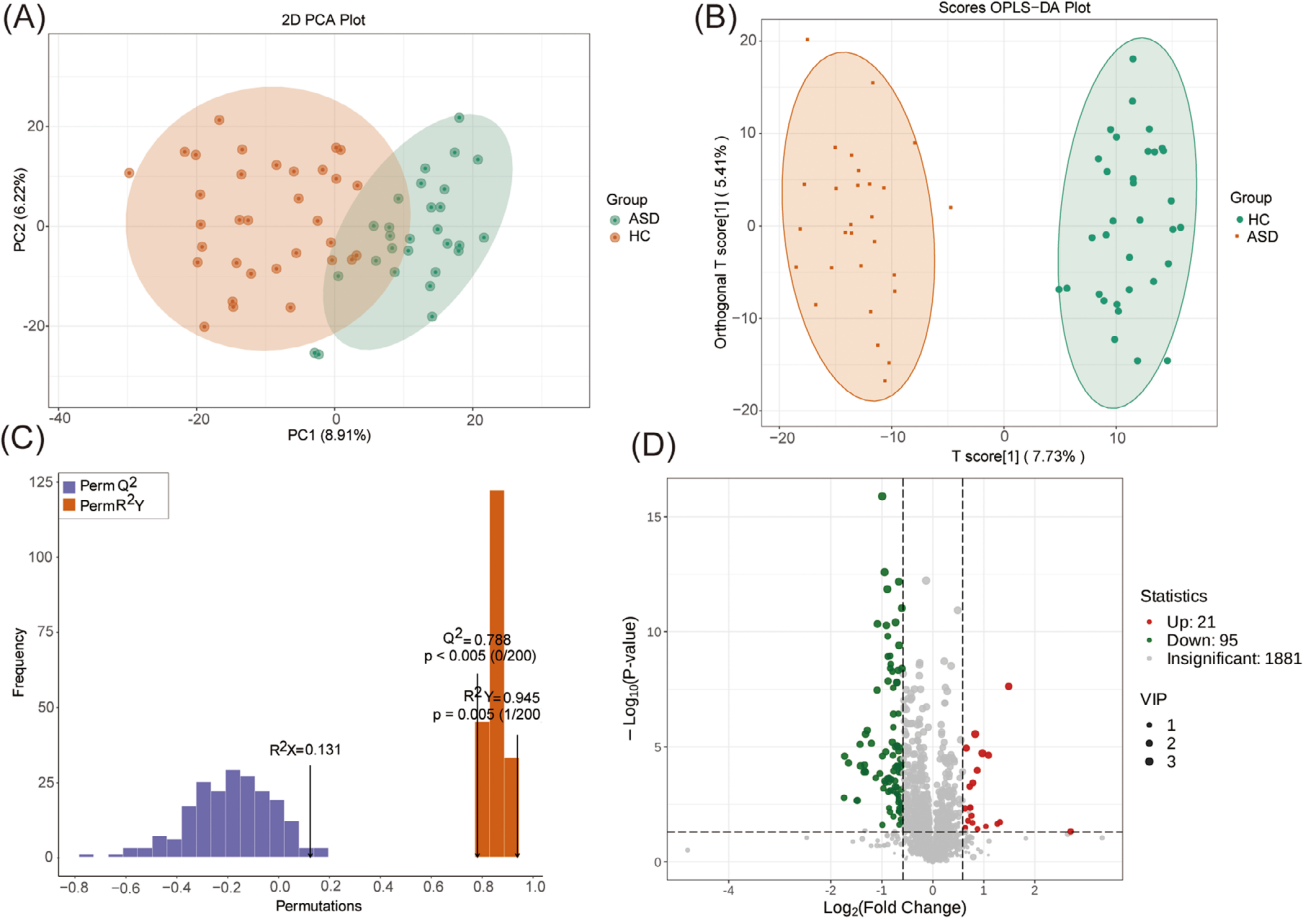
Identification of differential metabolites in the blood of healthy control (HC) and autism spectrum disorder (ASD). (A) Principal component analysis (PCA) between HC and ASD groups. Each data point corresponds to an individual sample, and the axes represent the principal components that capture the most significant variations in the dataset. The PCA analysis helps to assess the overall clustering patterns and potential group differences. (B) Orthogonal partial least squares discriminant analysis (OPLS‐DA) between HC and ASD groups. This plot showcases the discrimination between the HC and ASD groups using OPLS‐DA. It highlights the metabolic features responsible for driving the separation between the two groups. (C) The permutation test is utilized to evaluate the precision of the OPLS‐DA model. In this method, *R* ^2^ *X* and *R* ^2^ *Y* denote the proportion of variance explained by the model for the *X* and *Y* matrices, respectively, while *Q* ^2^ measures the model's predictive capability. (D) Volcano plot of differential metabolites between HC and ASD groups. This plot displays the fold change (on the *x*‐axis) versus the statistical significance (−log10 *p*‐value) (on the *y*‐axis) of the differential metabolites between HC and ASD groups.

The purpose of this analysis was to identify differential metabolites between the ASD and HC groups. To achieve this, several corresponding experiments were conducted using various statistical methods. First, principal component analysis (PCA) was employed as a dimensionality reduction method to evaluate the repeatability and variability of samples within and between groups based on their distances. The PCA results demonstrated a clear clustering pattern of samples within the ASD and HC groups, indicating potential metabolic profile differences between the two groups (Figure 3A). To further investigate the relationship between metabolite expression and subgroups, an orthogonal partial least squares discriminant analysis (OPLS‐DA) model was constructed. The OPLS‐DA score plot revealed distinct separation between the ASD and HC groups, indicating significant differences in their metabolic profiles (Figure 3B). The OPLS‐DA model was validated using a permutation assay, with *Q* ^2^ > 0.5 considered a robust model. The results indicated that the OPLS‐DA model had good stability and reliability, with *Q* ^2^ = 0.78, *R* ^2^ *Y* = 0.945, and *p* < 0.005 (Figure 3C). Based on the OPLS‐DA model, differential metabolites were identified by calculating the variable importance (VIP) value for each metabolite. The VIP value was combined with fold change (FC) and *p*‐values to determine the significance of differential expression. A total of 116 differential metabolites were identified, with 21 upregulated and 95 downregulated in the ASD group (Figure 3D). A detailed list of differential metabolites is provided in Table S1. These findings provide further evidence for the presence of metabolic differences between the ASD and HC groups.

### 2.4 Potential functional enrichment analysis of differential metabolites

**FIGURE 4:**
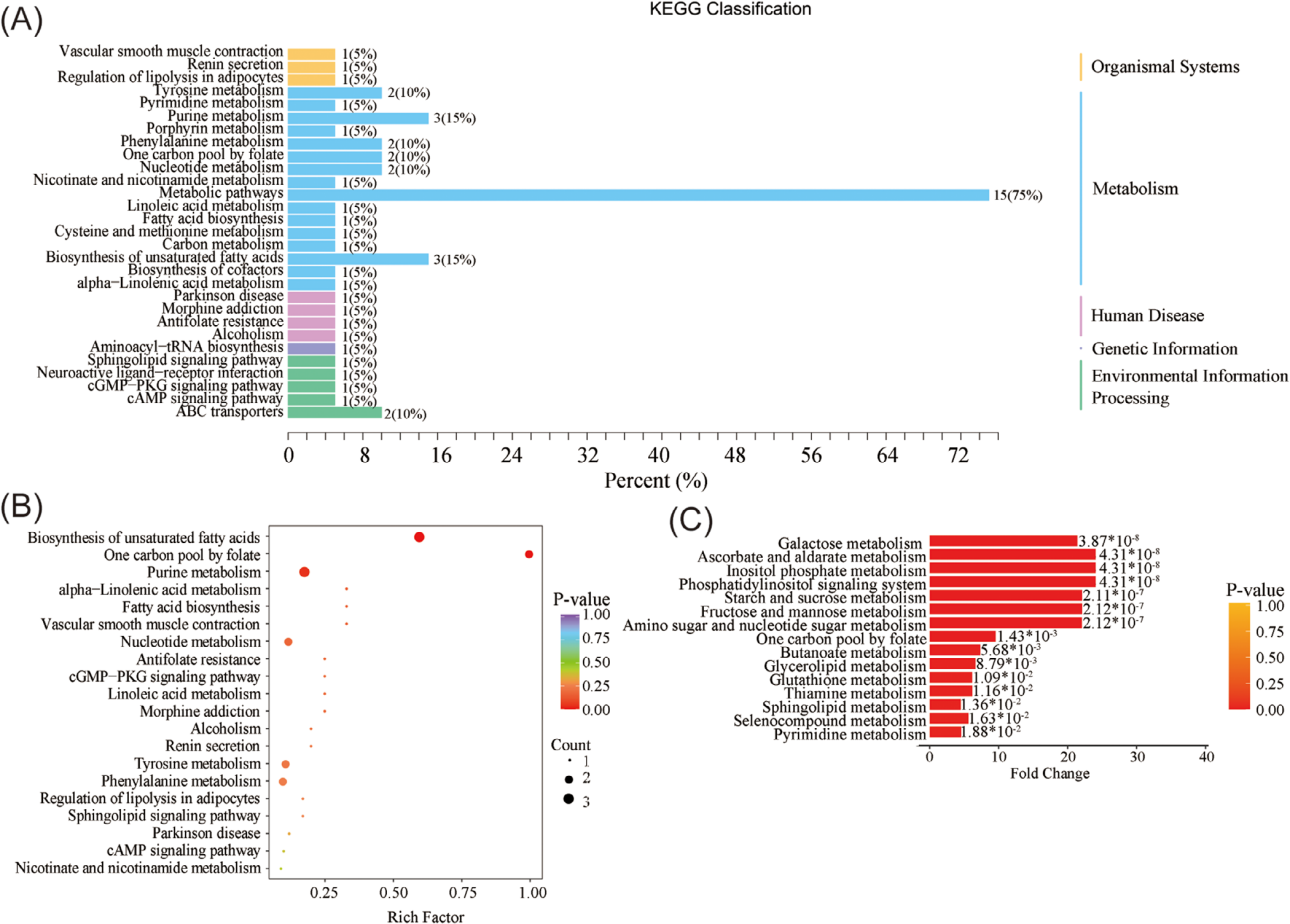
Functional enrichment analysis of differential metabolites. (A) Sorting the outcomes of Kyoto Encyclopedia of Genes and Genomes (KEGG) enrichment analysis by pathway classification. This visualization categorizes the results of the KEGG enrichment analysis based on pathway classification. It provides an organized overview of the enriched pathways and their corresponding biological functions. The pathways are grouped into different categories, facilitating a comprehensive understanding of the functional implications of the identified differential metabolites. (B) KEGG enrichment analysis of differential metabolites. Each pathway is represented by a dot, indicating its significance and impact. This information enables a deeper exploration of the biological processes and pathways involved in the observed metabolic differences between healthy control (HC) and autism spectrum disorder (ASD) groups. (C) Metabolic set enrichment analysis (MSEA) between HC and ASD groups. MSEA obviates the need for explicit differential metabolite thresholds by establishing a collection of metabolic sets, each corresponding to a specific biological function. Subsequently, the metabolomic data are enriched against these metabolic sets, and a statistical analysis is conducted to identify the significantly divergent metabolic sets.

In order to investigate the potential roles and pathways linked to the differential metabolites identified between individuals with ASD and those in the HC group, we conducted Kyoto Encyclopedia of Genes and Genomes (KEGG) enrichment analysis and metabolic set enrichment analysis (MSEA). In the KEGG enrichment analysis, the differential metabolites were categorized based on their associated pathways. The KEGG classification revealed that these metabolites were predominantly associated with metabolic pathways, purine metabolism, and biosynthesis of unsaturated fatty acids (Figure 4A).

Further KEGG enrichment analysis was performed to gain more detailed insights into the specific pathways enriched by the differential metabolites. The results showed that these metabolites were mainly enriched in unsaturated fatty acid biosynthesis, purine metabolism, nucleotide metabolism, folate metabolism, and linolenic acid metabolic pathways (Figure 4B). These findings suggest that dysregulation of metabolic pathways involved in lipid and nucleotide metabolism may be associated with ASD. To further elucidate the metabolic pathways that differed between the ASD and HC groups, MSEA was conducted. The MSEA results highlighted several significantly different pathways, including galactose metabolism, ascorbate and aldarate metabolism, inositol phosphate metabolism, phosphatidylinositol signaling system, and starch and sucrose metabolism (Figure 4C). These findings provide valuable insights into the underlying biological mechanisms associated with the observed differences in metabolite profiles between the ASD and HC groups. They may also serve as a basis for future studies examining the metabolic aspects of ASD.

### 2.5 Identification of potential diagnostic biomarkers for ASD

**FIGURE 5:**
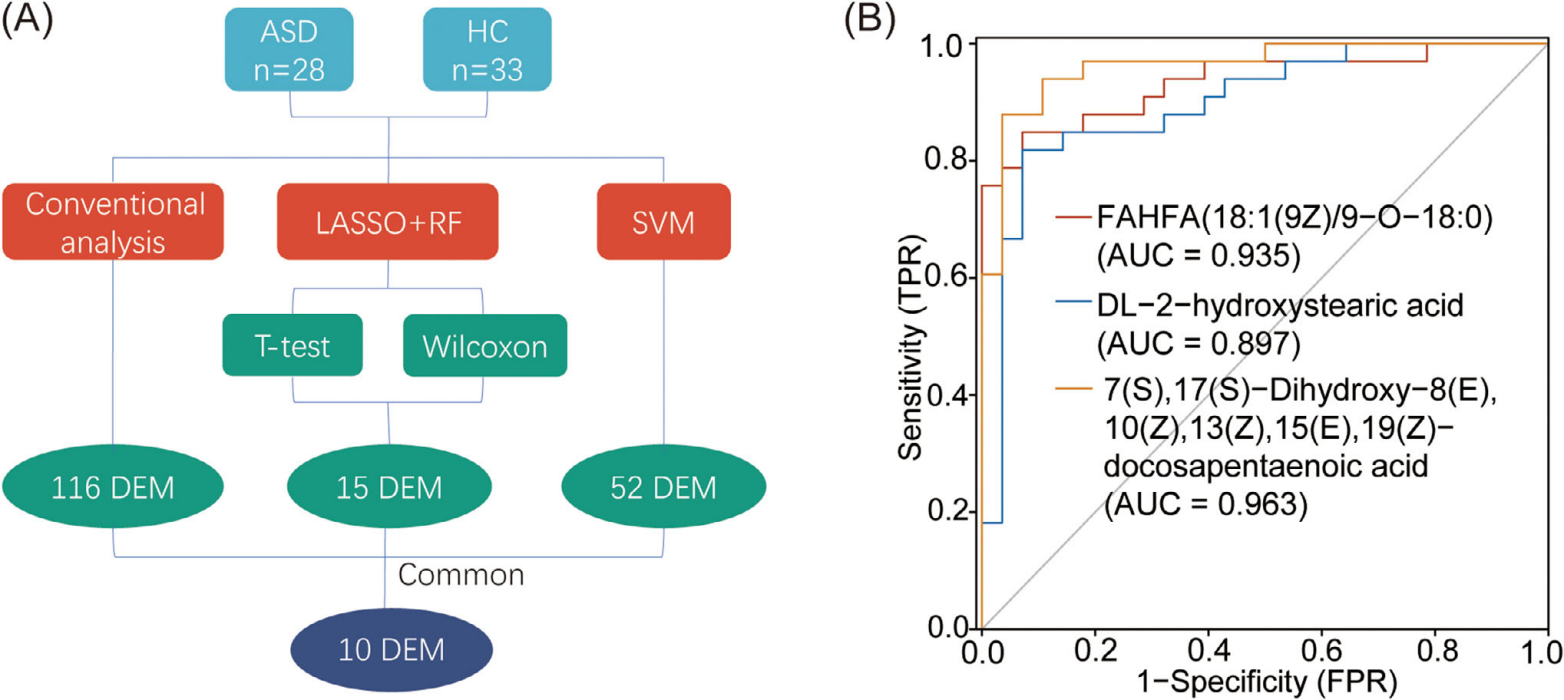
Identification of potential diagnostic biomarkers. (A) Flowchart for identifying potential biomarkers. This flowchart outlines the step‐by‐step process used to identify potential diagnostic biomarkers. (B) Receiver operating characteristic (ROC) analysis for potential markers. This plot displays the results of ROC analysis conducted to evaluate the diagnostic performance of the potential biomarkers. The *x*‐axis represents the false‐positive rate (1 − specificity), while the *y*‐axis represents the true‐positive rate (sensitivity). ASD, autism spectrum disorder; AUC, area under curve; HC, healthy control; LASSO, least absolute shrinkage selection operator; RF, random forest; SVM, support vector machine.

**FIGURE 6:**
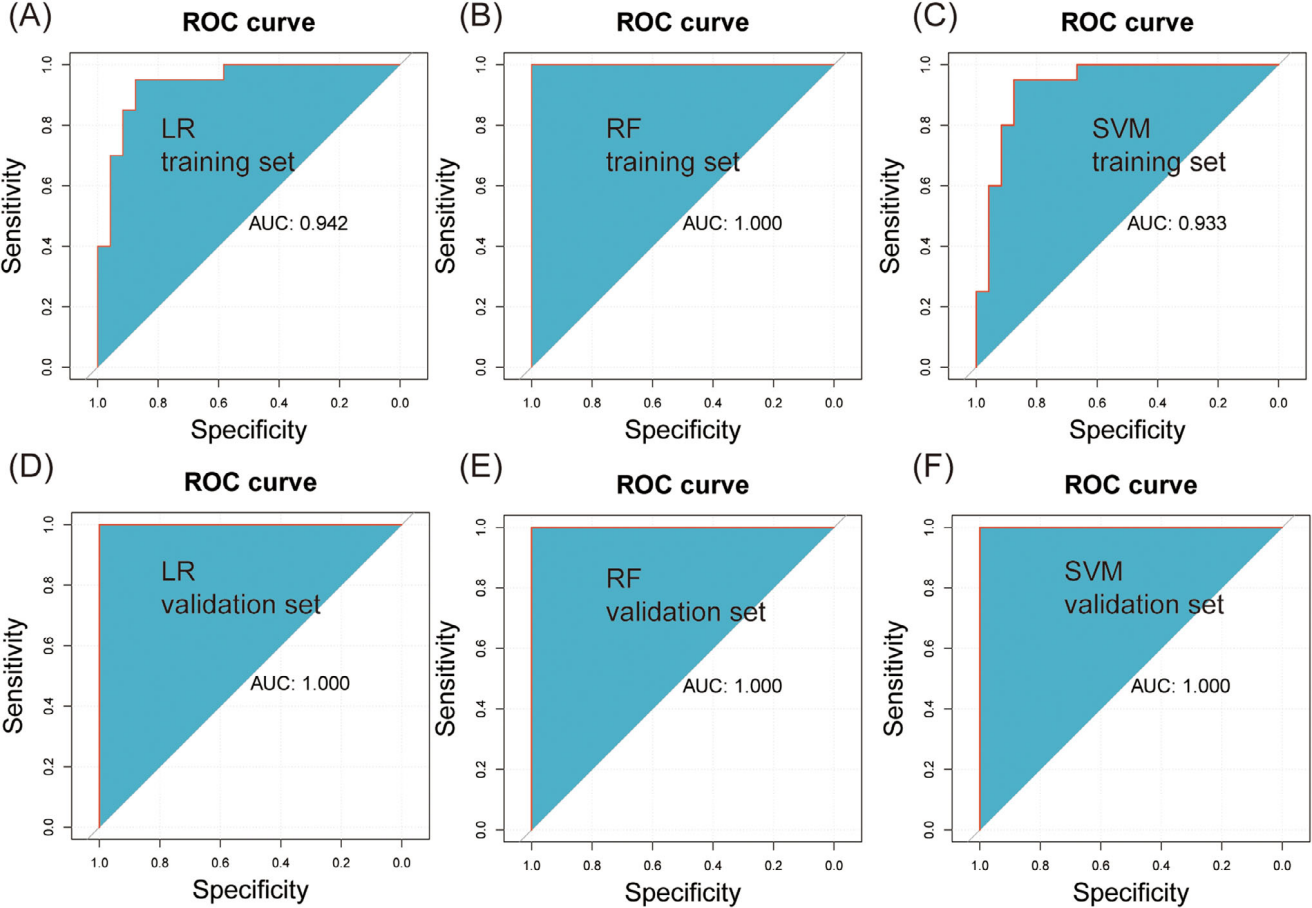
Construction and validation of diagnostic models. (A) Receiver operating characteristic (ROC) analysis of logistic regression (LR) models in the training set. This subfigure displays the performance of the LR models in distinguishing between autism spectrum disorder (ASD) and non‐ASD individuals within the training set. The *x*‐axis represents the false‐positive rate (1 − specificity), while the *y*‐axis represents the true‐positive rate (sensitivity). (B) ROC analysis of random forest (RF) models in the training set. A higher area under curve (AUC) value indicates superior performance in distinguishing between ASD and non‐ASD individuals. (C) ROC analysis of support vector machine (SVM) models in the training set. This subfigure showcases the ROC analysis outcomes for the SVM models in the training set. The AUC value quantifies the accuracy of these models in classifying ASD cases. (D) ROC analysis of LR models in the validation set. (E) ROC analysis of RF models in the validation set. (F) ROC analysis of SVM models in the validation set. The ROC curve illustrates the ability of the LR models to accurately classify individuals with and without ASD, specifically in the validation dataset.

To identify metabolic biomarkers with diagnostic potential for ASD, various machine learning algorithms were used to screen for characteristic metabolites. First, a total of 116 differential metabolites were initially screened based on VIP, *p*‐value, and FC. Second, least absolute shrinkage selection operator (LASSO) regression and random forest (RF) analysis with parametric test (*t*‐test) and nonparametric test (rank sum analysis) identified 16 and 15 differential metabolites, respectively, with 15 common differential metabolites identified by both statistical methods. Third, the 61 samples were then divided, in a 7:3 ratio, into 44 samples in the training set and 17 samples in the validation set, and the markers were screened using the training set. The screening process was as follows: log2 processing of the data followed by *z* score normalization; prescreening using FC < 2/3 or FC > 3/2, *p* < 0.05; correlation analysis of the results of the prescreening, with only one metabolite retained for pairs with correlation >0.8; LASSO screening, with the top 40 substances of VIP retained for subsequent screening; and the 40 metabolites were subjected to support vector machine (SVM) analysis. The final screening resulted in 40 differential metabolites, plus 12 metabolites were removed from the correlation analysis, so that 52 differential metabolites were obtained. The 116 differential metabolites obtained from conventional analysis, 15 metabolites obtained from LASSO and RF analysis, and 52 metabolites obtained from SVM analysis were intersected to finally obtain 10 common differential metabolites. To identify potential diagnostic biomarkers for ASD, 116 differential metabolites obtained from conventional analysis, 15 metabolites obtained from LASSO and RF analysis, and 52 metabolites obtained from SVM analysis were intersected to obtain 10 common differential metabolites (Figure 5A). Exogenous metabolites were excluded, resulting in a total of 3 candidate markers: FAHFA (18:1(9Z)/9‐O‐18:0), DL‐2‐hydroxystearic acid, and 7(S),17(S)‐dihydroxy‐8(E),10(Z),13(Z),15(E),19(Z)‐docosapentaenoic acid. ROC curve analysis showed good diagnostic performance in differentiating between children with ASD and HC, with area under curves (AUCs) of 0.935, 0.897, and 0.963 for the three candidate markers, respectively (Figure 5B). To clarify, the final three candidate markers (FAHFA (18:1(9Z)/9‐O‐18:0), DL‐2‐hydroxystearic acid, and 7(S),17(S)‐dihydroxy‐8(E),10(Z),13(Z),15(E),19(Z)‐docosapentaenoic acid) were used to construct logistic regression, RF, and SVM models, which yielded high AUC values for both the training set (0.942, 1.000, and 0.933, respectively) (Figure 6A–C) and validation set (1.000, 1.000, and 1.000, respectively) (Figure 6D–F). These findings suggest that these metabolites may serve as potential diagnostic biomarkers for ASD.

## 3 DISCUSSION

The prevalence of ASD is increasing globally, highlighting the need for early diagnosis and intervention.^21^ However, the lack of early symptoms and limitations in current diagnostic methods pose challenges in this regard. Therefore, identifying specific biomarkers for ASD that enable early diagnosis and intervention holds significant clinical and research value.

Metabolomics is indeed a powerful tool for studying the metabolites in organisms, and its application in ASD research can provide valuable insights into the underlying pathological mechanisms of the disorder.^22^ By comparing the differences in metabolite quantities and types between normal individuals and those with ASD, we can identify potential biomarkers that may be associated with the condition. This information can be useful for the early diagnosis of ASD and the development of more effective treatments. It is becoming increasingly clear to researchers that metabolic disorders may be an underlying cause of ASD.^23^ Dan et al. reported significant abnormalities in the metabolic levels of fatty acids, nucleotides, and amino acids, which are associated with the metabolism of neurotransmitters, in children with ASD compared to normally developing children.^24^ Kelly et al. found significant dysregulation of the pathways of tryptophan biosynthesis, tyrosine metabolism, and endogenous cannabinoid metabolism in children with ASD.^25^ These findings have important implications for our understanding of ASD and its underlying mechanisms. By identifying specific biomarkers and metabolic pathways associated with the disorder, we can potentially improve diagnostic and treatment options for affected individuals. For instance, targeting these pathways through dietary interventions or pharmacological treatments may help to alleviate symptoms or delay the onset of the disorder. However, more research is needed to fully understand the complex relationship between metabolic disorders and ASD. Future studies should explore the exact mechanisms underlying these differences in metabolic profiles and investigate potential risk factors such as genetics and environmental exposures. Ultimately, a better understanding of these factors may lead to improve prevention, early intervention, and treatment strategies for ASD. In this study, a total of 116 metabolites that exhibited differential expression between the ASD and HC groups were identified using metabolomics. The majority of these metabolites belong to the categories of amino acids and their metabolites, followed by organic acids. Further analysis using the KEGG revealed that these 116 differentially expressed metabolites were predominantly enriched in pathways related to unsaturated fatty acid biosynthesis, purine metabolism, nucleotide metabolism, folate metabolism, linolenic acid metabolic pathways, among others. These results showed significant differences in metabolic profiles between ASD children and HC subjects, which may provide clues for further understanding of the role of relevant metabolic pathways in the development of ASD.

The three differential metabolites obtained in this study were all fatty acids. Several studies have shown that abnormalities in fatty acid metabolism may be associated with an increased risk of developing ASD.^16^, ^26^, ^27^ Long‐chain polyunsaturated fatty acids such as arachidonic acid and docosahexaenoic acid, which are the most biologically active compounds in the fatty acid family, may influence neural responses and behavior, and their concentrations in the brain may vary considerably.^28^, ^29^, ^30^, ^31^ Polymorphisms in genes involved in mevalonate/cholesterol metabolism have also been associated with an increased risk of developing ASD.^32^ Cholesterol is transported through lipoproteins in the blood with fatty acid esterification, highlighting the close relationship between fatty acid metabolism and cholesterol metabolism.^33^, ^34^ Furthermore, a retrospective population‐based study showed that certain fatty acid‐related biomarkers were positively associated with late diagnosis of ASD, while others were negatively correlated with late diagnosis of ASD.^35^ These findings suggest that dysregulation of fatty acid metabolism may play a role in the development of the disorder. However, the exact mechanisms underlying the link between fatty acid metabolism and ASD still require further investigation. Future research should aim to elucidate the specific metabolic pathways and genes involved in this relationship, as well as potential environmental factors such as diet and toxin exposure that may contribute to metabolic dysregulation. This information may lead to new preventative and treatment strategies for ASD.

However, there are some other limitations to this study. First, the sample size was relatively small, with only 28 children with ASD and 33 HC included. This small sample size may limit the generalizability of the findings and increase the possibility of selection bias. Replication of these results in a larger cohort would add more robustness to the conclusions. Additionally, although the selected metabolites showed promising discriminatory efficacy, it is important to note that they were identified based on plasma samples alone. Further investigations using other biological matrices such as urine or cerebrospinal fluid could provide a more comprehensive understanding of the metabolic alterations associated with ASD. Another limitation is the lack of information regarding potential confounding factors such as age, sex, and medication use among the participants. These factors can influence metabolic profiles and may introduce additional variability in the results. Adjusting for these factors or performing subgroup analyses based on relevant characteristics could enhance the reliability and applicability of the findings. Furthermore, the diagnostic models constructed in this study need to be externally validated using an independent cohort. Cross‐validation within the same dataset may overestimate the model's performance, and external validation is necessary to assess its generalizability and clinical utility. Lastly, while this study provides valuable insights into potential metabolic markers for ASD diagnosis, further functional studies are needed to elucidate the underlying mechanisms and validate the causal relationship between these metabolites and ASD pathophysiology.

This study utilized the UPLC‐MS/MS platform and widely targeted metabolic technology to characterize the metabolic profile of ASD and compared it with HC subjects. The findings revealed significant changes in various metabolic pathways, including unsaturated fatty acid biosynthesis, purine metabolism, nucleotide metabolism, folate metabolism, and linolenic acid metabolic pathways. These results suggest that the identified metabolome biomarkers, such as FAHFA (18:1(9Z)/9‐O‐18:0), DL‐2‐hydroxystearic acid, and 7(S),17(S)‐dihydroxy‐8(E),10(Z),13(Z),15(E),19(Z)‐docosapentaenoic acid, could potentially be used for the diagnosis of ASD. Furthermore, these biomarkers may serve as targets for new treatment strategies aimed at reducing the high mortality rate associated with ASD. Overall, this study provides valuable insights into the metabolic alterations associated with ASD and offers potential avenues for improving children care.

## 4 MATERIALS AND METHODS

### 4.1 Sampling and ethics

In this study, the design and procedures were in line with the Declaration of Helsinki and approved by the Ethics Committee of Yue Bei People's Hospital, Shantou University Medicine College (China, KY‐2022‐077). Written informed consent was obtained from all parents of the 61 participants, including 28 children with ASD and 33 healthy controls, from Shaoguan Maternal and Child Health Hospital and Renhua County Maternal and Child Health Hospital. All ASD subjects were diagnosed by clinical experts from the Department of Pediatric Rehabilitation of Shaoguan Maternal and Child Health Hospital based on the Diagnostic and Statistical Manual of Mental Disorders V. Children who had childhood schizophrenia, purely psychotic developmental disorders, simple language developmental disorders, other pervasive developmental disorders, deafness, organic neurological disorders, heart, liver, or kidney diseases, inflammatory or infectious diseases, and those taking medication during the study period were excluded.

### 4.2 Sample collection and extraction

The same brand and type of anticoagulation tubes were used to collect the samples, which were processed in the same laboratory using the same procedures and equipment. For each child, approximately 3 mL of whole blood was collected under fasting conditions using an EDTA anticoagulant tube. The plasma was obtained by centrifuging the blood at 3000 rpm for 10 min at 4°C. The plasma was separated in a biosafety cabinet and stored at −80°C. To prepare the samples for analysis, they were thawed completely in an ice box and vortexed for 10 s to mix thoroughly. Then, 50 µL of the sample was transferred to a centrifuge tube, and 300 µL of metabolite extract containing 20% acetonitrile–methanol internal standard was added to the tube. After vortexing for 3 min, the tube was centrifuged at 12,000 r/min for 10 min at 4°C. The supernatant (200 µL) was transferred to another corresponding numbered centrifuge tube and left for 30 min at −20°C in the refrigerator. Subsequently, the tube was centrifuged at 12,000 r/min for 3 min at 4°C, and 180 µL of the supernatant was transferred to the liner tube of the corresponding injection vial and used for TM widely targeted analysis. The TM widely targeted was used in this project, and the TM widely targeted was a combination of a nontargeted and a widely targeted.

### 4.3 Metabolomics study

#### 4.3.1 T3 UPLC conditions

The primary component of the data acquisition instrumentation system was the ultraperformance liquid chromatography (UPLC) instrument (ExionLC AD, available at https://sciex.com.cn/). The liquid chromatography conditions used for this study were as follows, column size: ACQUITY HSS T3 (2.1 × 100 mm, 1.8 um), mobile phase A configuration: 0.1% formic acid/water, mobile phase B configuration: 0.1% formic acid/ethanide, flow rate set to 0.4 mL/min, injection volume set to 5 uL; positive and negative ion mobile phase gradient conditions: 95% mobile phase A, 5% mobile phase B at 0 min, 10% mobile phase A, 90% mobile phase B at 11 min, 10% mobile phase A, 90% mobile phase B at 12 min, 95% mobile phase A, 90% mobile phase B at 12.1 min, positive and negative ion mobile phase gradient conditions: 95% mobile phase A, 5% mobile phase B at 0 min, 10% mobile phase A, 90% mobile phase B at 12 min, 95% mobile phase A, 90% mobile phase B at 12.1 min. mobile phase A, 5% of mobile phase B at 0 min, 10% of mobile phase A, 90% of mobile phase B at 11 min, 10% of mobile phase A, 90% of mobile phase B at 12 min, 95% of mobile phase A, 5% of mobile phase B at 12.1 min, 95% of mobile phase A, 5% of mobile phase B at 14 min.

#### 4.3.2 Quadrupole‐Time of Flight‐MS/MS

The Quadrupole‐Time of Flight Mass Spectrometry instrument (TripleTOF 6600, manufactured by AB SCIEX) was used to collect and process MS/MS spectra and to evaluate MS data from full‐scan measurements. The mass spectrometry conditions mainly include, positive and negative ion mode mass spectrometry conditions: curtain gas (electrospray ionization (ESI)+:25, ESI−:25), ionspray voltage (ESI+:5500, ESI−:4500), temperature (ESI+:500, ESI−:500), ion source gas 1 (ESI+:50, ESI−:50), ion source gas 2 (ESI+:50, ESI−:50), declustering potential (ESI+:80, ESI−:−80), collision energy (ESI+:30, ESI−:−30), collision energy spread (ESI+:15, ESI−:15).

#### 4.3.3 ESI‐QTRAP‐MS/MS

The triple quadrupole (QQQ) scan was acquired using a QQQ‐linear ion trap mass spectrometer (QTRAP), LC‐MS/MS system of the QTRAP series, with an ESI Turbo Ion‐Spray interface, operated in positive and negative ion modes, and controlled by the Analyst 1.6.3 software (Sciex). In this study, the electrospray ionization temperature was set at 500°C. The mass spectrometry voltage was set to 5500 V in positive mode and −4500 V in negative mode. The ion source gas I and gas II were both set to 50 psi, and the curtain gas was set to 25 psi. Collision‐activated dissociation parameter was set to high. In the QQQ, each ion pair was scanned and detected using the optimized declustering potential and collision energy.

### 4.4 Metabolomics data analysis

In this study, the qualitative analysis of metabolites was performed by comparing the downstream data with multiple databases. This involved searching for known metabolites and matching them to the acquired data based on their mass‐to‐charge ratio and other relevant features. The quantitative analysis of metabolites, on the other hand, was conducted using the multiple reaction monitoring (MRM) mode of QQQ mass spectrometry. The triple quadruple mass spectrometry was employed to filter out the characteristic ions of each substance, and the detector measured the signal intensity of these ions. The MultiQuant software was utilized to access the downstream mass spectrometry file, perform chromatographic peak integration and calibration, and determine the relative content of each substance based on the peak area of each chromatographic peak. Finally, all the data regarding the integration of chromatographic peak areas were exported and saved. MRM is a targeted technique that involves selecting specific precursor ions in the first quadrupole, fragmenting them in the collision cell, and then selectively detecting certain product ions in the third quadrupole. By monitoring these transitions, it is possible to quantify the abundance of specific metabolites in the sample. The use of MRM allowed for more precise and accurate measurement of metabolite concentrations compared to the qualitative analysis.

### 4.5 Bioinformatics and statistical analysis

Data analysis and graphs were generated using R software version 4.1.1 in this study. The characteristic ions of each metabolite were monitored using multiple reactions of the mesoftware database. Chromatographic review and peak area integration analysis were conducted using MultiQuant software. PCA is an unsupervised statistical analysis technique used in pattern recognition for multidimensional data. It aims to transform a set of potentially correlated variables into a new set of linearly uncorrelated variables through an orthogonal transformation. The samples underwent PCA using the ggplot package to obtain an initial comprehension of the overall disparities in metabolites among sample groups and the extent of variability within sample groups. The resulting set of variables is referred to as principal components. Projected VIP was calculated based on OPLS‐DA and combined with *p*‐values from *t*‐tests and FC to identify differential metabolites. The criteria used for screening differential metabolites were VIP value >1, *p* < 0.05, FC > 1.5 or FC < 0.67. The OPLS‐DA method, which integrates the orthogonal signal correction and partial least squares‐discriminant analysis (PLS‐DA) techniques, enables the decomposition of the *X* matrix information into two distinct categories: one that is correlated to *Y* and another that is uncorrelated. Additionally, it effectively filters out the uncorrelated differences by removing them from the variance variables. The analysis was conducted using the OPLSR.Anal function from the MetaboAnalyst R package in the R software. The functions of the enriched differential metabolites were annotated with KEGG, a widely used database for pathway analysis. This allowed for the identification of metabolic pathways that were most affected by the observed changes in metabolite concentrations. Overall, these analytical methods enabled a comprehensive and systematic analysis of the metabolomic data generated in this study, providing insights into the underlying biological mechanisms associated with the observed changes in metabolites. Potential biomarkers were screened by LASSO, SVM, and RF. The study population was split into a training set and a validation set at a ratio of 7:3. Diagnostic models were developed using logistic regression, SVM, RF in the training set and subsequently evaluated in the validation set.

## AUTHOR CONTRIBUTIONS

J.L., Y.T., and F.Z.: Writing of the article, serum collection, and data analysis. Y.T., Y.W., S.C., and N.Z.: Diagnosis and the follow up of patients. W.D. and L.Z.: Serum collection and data analysis. J.‐C. L.: Study design, writing‐review, and editing. All the authors have read and approved the final manuscript.

## CONFLICT OF INTEREST STATEMENT

The authors declare that there are no conflicts of interest.

## ETHICS STATEMENT

The study design and protocol were ethically approved by the ethics committee of the Yue Bei People's Hospital affiliated to Shantou University Medical College (Shaoguan, China), No: KY‐2022‐077. Written informed consent was obtained from all participants.

## Supporting information

Supplement Table 1: Basic information on differential metabolites

## Data Availability

The original data are available on request from the corresponding author.
